# CD161^+^ MAIT Cells Are Severely Reduced in Peripheral Blood and Lymph Nodes of HIV-Infected Individuals Independently of Disease Progression

**DOI:** 10.1371/journal.pone.0111323

**Published:** 2014-11-04

**Authors:** Johanna Maria Eberhard, Philip Hartjen, Silke Kummer, Reinhold E. Schmidt, Maximilian Bockhorn, Clara Lehmann, Ashwin Balagopal, Joachim Hauber, Jan van Lunzen, Julian Schulze zur Wiesch

**Affiliations:** 1 Infectious Diseases Unit, Department of Medicine, University Medical Center Hamburg-Eppendorf, Hamburg, Germany; 2 Department of Oral and Maxillofacial Surgery, University Medical Center Hamburg-Eppendorf, Hamburg, Germany; 3 Department of General Surgery, University Medical Center Hamburg-Eppendorf, Hamburg, Germany; 4 Heinrich Pette Institute - Leibniz Institute for Experimental Virology, Hamburg, Germany; 5 Department of Immunology and Rheumatology, Hannover Medical School, Hanover, Germany; 6 Department of Internal Medicine I, University Hospital of Cologne, Cologne, Germany; 7 School of Medicine, Johns Hopkins University, Baltimore, Maryland, United States of America; 8 German Center for Infection Research (DZIF), partner site Hamburg and Hannover, Hamburg and Hannover, Germany; Beth Israel Deaconess Medical Center, Harvard Medical School, United States of America

## Abstract

Mucosal-associated invariant T (MAIT) cells are characterized by the combined expression of the semi-invariant T cell receptor (TCR) Vα7.2, the lectin receptor CD161, as well as IL-18R, and play an important role in antibacterial host defense of the gut. The current study characterized CD161^+^ MAIT and CD161^–^TCRVα7.2^+^ T cell subsets within a large cohort of HIV patients with emphasis on patients with slow disease progression and elite controllers. Mononuclear cells from blood and lymph node samples as well as plasma from 63 patients and 26 healthy donors were analyzed by multicolor flow cytometry and ELISA for IL-18, sCD14 and sCD163. Additionally, MAIT cells were analyzed after *in vitro* stimulation with different cytokines and/or fixed *E.coli*. Reduced numbers of CD161^+^ MAIT cells during HIV infection were detectable in the blood and lymph nodes of all patient groups, including elite controllers. CD161^+^ MAIT cell numbers did not recover even after successful antiretroviral treatment. The loss of CD161^+^ MAIT cells was correlated with higher levels of MAIT cell activation; an increased frequency of the CD161^–^TCRVα7.2^+^T cell subset in HIV infection was observed. *In vitro* stimulation of MAIT cells with IL-18 and IL-12, IL-7 and fixed *E.coli* also resulted in a rapid and additive reduction of the MAIT cell frequency defined by CD161, IL-18R and CCR6. In summary, the irreversible reduction of the CD161^+^ MAIT cell subset seems to be an early event in HIV infection that is independent of later stages of the disease. This loss appears to be at least partially due to the distinctive vulnerability of MAIT cells to the pronounced stimulation by microbial products and cytokines during HIV-infection.

## Introduction

Chronic untreated HIV infection is characterized by general immune activation, immune dysregulation, high T cell turnover and a gradual decline of CD4^+^ T cells through infection and bystander activation induced apoptotic death [1]. The translocation of microbial products from the gastrointestinal (GI) tract to portal and systemic circulation has been proposed as a major driver of the generalized chronic immune activation that is associated with HIV disease progression [2].

A recently described T cell subset with limited receptor diversity and high abundance in mucosal tissues has been shown to recognize microbial products. These cells, termed mucosal-associated invariant T (MAIT) cells, can be identified by the surface expression of CD161 and the invariant TCRVα7.2 segment [1]. In general, MAIT cell responses are restricted by the conserved MHC-related-molecule-1 (MR1) that presents riboflavin precursors derived from bacteria and yeasts predominantly in the gut [3]. The MAIT cell defining surface marker CD161 is a C-type lectin-like membrane receptor that can bind its ligand, the lectin-like transcript 1 (LLT1), with yet unclear function [4], [5]. MAIT cells exhibit a tissue-targeting memory phenotype and express high levels of cytokine receptors for IL-18, IL-12 and IL-23 [4], [6]–[8]. Moreover, MAIT cells exhibit specific effector activities such as TNF-α, IFN-γ, IL-17 production as well as granzyme B secretion [4], [6], [7].

Recent reports describe a significant loss of CD161^+^ MAIT cells from the circulation of HIV- infected patients [9]–[12]. It is thought that the decrease of these cells not only weakens the defense against bacterial pathogens like *Escherichia coli*, *Candida albicans* and *Mycobacterium tuberculosis* (MTB) [1]–[4], but could also further enhance the intestinal translocation of microbial products, which is related to the chronic activation and exhaustion of the immune system associated with HIV-infection [2], [6], [9], [11], [12]. However, the timing, kinetics and mechanisms behind the reduction of peripheral CD161^+^ MAIT cells in patients with HIV-infection and different disease course has not been fully elucidated. On the one hand, activation induced cell death and accumulation in tissues could explain the severe reduction of MAIT cells. On the other hand a change of phenotype in terms of down-regulation of marker molecules such as CD161 following activation and exhaustion have been suggested as alternative explanation [5]–[7]. Limited data on the tissue distribution of MAIT cells are available so far, especially with regard to secondary lymphoid tissues. Furthermore, there are extant questions about the early kinetics of MAIT cells during acute infection, and whether patients who exhibit natural control of HIV have spared MAIT cells [8] [11]. In particular, the loss and exhaustion of MAIT cells could have important implications for the mucosal defense against bacterial pathogens in the gut of HIV-infected patients. Latest studies contradict earlier assumptions [9] and show that HIV elite controllers (EC) show activated innate immune responses in comparison to healthy controls despite controlling plasma HIV viremia [13]–[15]; therefore, these results are consistent with an impaired mucosal barrier.

In the current study, MAIT cells, defined as CD161^+^ TCR Vα7.2^+^ CD4^–^ T cells as well as CD161^–^TCR Vα7.2^+^ CD4^–^ T cells are characterized *ex vivo* and after *in vitro* stimulation in a large cohort of HIV patients with emphasis on patients with slow disease progression and elite controllers (EC). This study aimed to gain further insight into the kinetics as well as possible mechanisms of MAIT cell decline during HIV-infection including the important compartment of lymphoid tissue.

## Results

### CD161^+^ MAIT cells are severely reduced in the blood and lymph nodes of HIV-infected patients independently of their disease status

The aim of this study was to comprehensively analyze MAIT cell subsets in the peripheral blood as well as in the lymph node compartment of a large cohort of patients with different HIV disease status.

**Figure 1:**
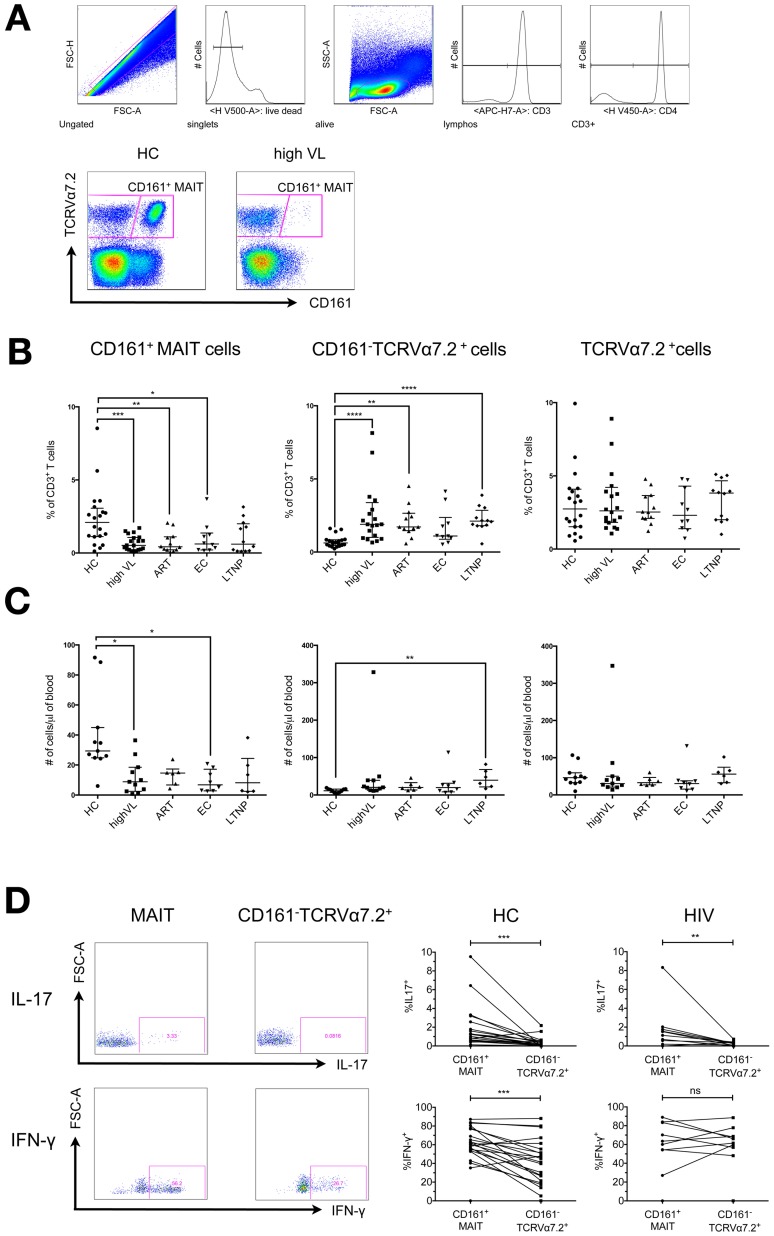
CD161^+^TCRVα7.2^+^ MAIT cells are severely reduced in the blood of HIV-infected patients. **A**) Gating Strategy for CD161 and TCRVα7.2 based MAIT cell characterization in the CD4^–^CD3^+^T cell subset. All FACS analyses were performed on frozen PBMC samples. Depicted are representative plots from healthy control PBMCs (upper panel). Gating of the MAIT cell subsets comparing a healthy donor (HC) and an HIV patient (lower panel). **B**) Cumulative display of CD161^+^TCRVα7.2^+^ MAIT cell frequencies, CD161^–^TCRVα7.2^+^ and total TCRVα7.2^+^ cells in % of CD3^+^ T cells comparing PBMC samples derived from healthy controls (HC n = 20), untreated patients with high viral loads (high VL n = 20), patients receiving antiretroviral treatment for a at least one year (ART n = 12), elite controller (EC n = 10) and patients with very low viremia and slow progression (<5000 copies/ml; LTNP n = 12). **C**) Absolute MAIT cell counts, CD161^–^TCRVα7.2^+^ and total TCRVα7.2^+^ cells per µl peripheral blood, calculated based on absolute CD3^+^ T cell counts (available for 12 HC, 11 high VL, 6 ART, 8 EC and 6 LTNP). All groups were tested for normal distribution with the Kolmogorov-Smirnov test and were compared by the adequate test, ANOVA followed by Tukey's multiple comparisons test or Kruskal-Wallis followed by multiple comparison test, respectively. P-values smaller than 0.05 were considered significant, where *, ** and *** indicate p-values between 0.01 to 0.05, 0.001 to 0.01 and 0.0001 to 0.001, respectively. Bars and lines indicate median, 25% and 75% quartiles. **D**) Representative FACS plots and the percentages of cells that produce the cytokines IL-17 and INF-γ upon stimulation with PMA/ionomycin among MAIT cells and CD161^–^TCRVα7.2^+^ cells are shown. PBMC samples derived from healthy donors (n = 22) and HIV-infected patients (ART treated and untreated with high viral loads, n = 10) were analyzed. Asterisks indicate the results of Wilcoxon matched-pairs signed-rank tests.

We first determined the frequency of CD161^+^ MAIT cells in the peripheral blood in a cohort of healthy, HIV-negative donors (Fig. 1). The vast majority of the CD3^+^CD161^+^TCRVα7.2^+^ MAIT cell population (>90%) either expresses CD8αα or CD8αβ, whereas a smaller subset (<10%) is CD8^–^CD4^–^ and only a negligible fraction of these cells expresses the CD4 molecule [10]. For simplicity and for better comparability with previous studies we defined MAIT cells as CD161^+^TCRVα7.2^+^CD4^–^CD3^+^ T cell population. This gating strategy allowed us to exclude the effect of general CD4^+^ T cell depletion from the analysis. Our gating strategy is depicted in Fig. 1A. Healthy controls displayed a broad range of MAIT cell frequencies, quantified in two ways: i) as percentage of CD3^+^ T cells (median = 2.1%, IQR = 1.12–3.06%) and ii) as percentage of CD4^–^ T cells (mean = 8.12%, IQR = 5.6–12.25%; data not shown) (Fig. 1B). This was reflected in a pattern of similar variation of the absolute number of CD161^+^ MAIT cells per µl of blood (Fig. 1C) and thus is not merely a consequence of varying parent population sizes. Our results are consistent with previous results from studies that determined MAIT cell frequencies in healthy subjects [5], [11], [12].

In accordance with previous studies [5]–[7], [13] we observed a severe reduction of the peripheral CD161^+^ MAIT cell subset of untreated and treated HIV-infected patients (median HC = 2.1%, IQR = 1.12–3.06% vs. median high VL = 0.52%, IQR = 0.22–1.06% and median ART = 0.42%, IQR = 0.22–1.1%, ANOVA followed by Tukey's multiple comparison test: p<0.001 and p<0.01, respectively) (Fig. 1B, C).

Importantly, we extended the results of the aforementioned studies by characterizing the frequency and function of MAIT cells in a group of elite controllers (n = 10) and long-term nonprogressors (n = 12) (Table 1) to answer the question whether spontaneous viral control or slower disease progression are associated with the preservation of peripheral MAIT cells. Throughout this study LTNPs were defined as: HIV Patients with low viremia (<5000 copies/ml) that maintain a favourable course of infection (CD4 counts>350 cells/µl) over 5 years in the absence of therapy.

**Table 1:** Cohort (PBMC samples).

	Healthy	high VL	ART	EC	LTNP
**number of donors**	20	21	12	10	12
**% female**	53.3	29.4	36.4	57.1	40
**age [years]**	39.2(±11.2)	39.9(±11.7)	48.9(±16)	46(±11.6)	43.92(±15.3)
**time since first diagnosis [month]**	–	51.2(±71.6)	101.2(±58.5)	150.5(±69.9)	106.8(±67.1)
**viral load [copies/ml]**	–	1580000(±3080000)	10 of 12: below detection limit; 2 of 12: <200	below detection limit	986.1 (±1655)
**CD4 [c/**µ**l]**	798.2(±308.8)	243.6(±289.1)	627.5(±363.1)	780.7(±284.7)	591.8(±176.8)
**CD8 [c/**µ**l]**	439(±154.1)	951.4(±980.8)	844.3(±266.7)	686.4(±305.9)	1217(±480.9)
**CD3 [c/**µ**l]**	1323(±418.8)	1594(±1471)	1475(±536.2)	1487(±588.1)	2140(±722.5)
**lymphocytes [c/**µ**l]**	1785(±527.7)	1613(±1325)	1997(±627.5)	2014(±712.5)	2386(±770)

Shown are means and standard deviation.

Interestingly, we observed a reduction of the CD161^+^ MAIT cell subset in both groups of patients (LTNP as well as EC) compared to healthy controls (median HC = 2.1%, IQR = 1.12–3.06% vs. median EC = 0.62%, IQR = 0.21–1.37% and median LTNP = 0.61, IQR = 0.14–2.0%) (Fig. 1B). This reduction was slightly less pronounced in EC and not statistically significant for LTNP (ANOVA followed by Tukey's multiple comparison test: p<0.05 for EC).

In previously published studies, it has been hypothesized that the disappearance of MAIT cells from the peripheral blood in chronic HIV infection might be best explained by a combination of activation induced cell death and/or homing of MAIT cells to different tissues and the gut [7]. Thus, in order to advance the understanding of possible tissue redistributions of MAIT cells in the course of the HIV infection, we extended our analysis by inclusion of lymph nodal MAIT cells. As natural killer T cells MAIT cells might have an important role in B cell help [14]–[16]. Previously, high expression of CCR6 and low expression of CCR7 have been observed on MAIT cells, indicating that these cells are mainly tissue circulating T cells [17].

**Figure 2:**
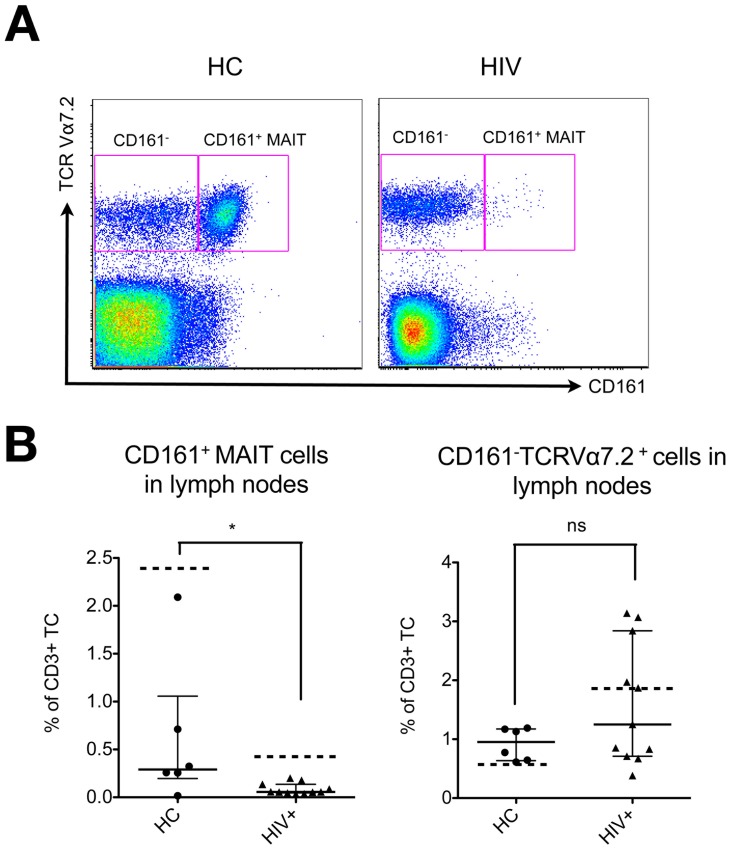
The frequency of CD161^+^ MAIT cells is severely reduced in lymph nodes of HIV-infected patients. **A**) Gating strategy for the characterization of MAIT cells and CD161^-^TCRVα7.2^+^ cells within the CD4^–^ T cell subset of lymph node mononuclear cells (LNMC). Representative FACS plots for LNMC from a healthy control and an HIV-infected patient are shown. **B**) Frequencies of CD161^+^ MAIT cells and CD161^–^TCRVα7.2^+^ cells as percentages among CD3^+^ lymphocytes for LNMC samples derived from healthy controls (healthy, n = 6) and HIV-infected patients (HIV, n = 11). Bars and lines indicate median and interquartile range. Dotted lines indicate the frequencies in the PBMC compartment of unmatched but similar groups for comparison. P-values are results of unpaired Mann-Whitney tests.

We also found that lymph node samples of healthy controls and HIV patients (Table 2) generally showed a significantly lower frequency of MAIT cells compared to PBMC of unpaired samples of the same groups (p<0.01 in HC and p<0.0001 in HIV patients, Mann-Whitney tests), while the frequencies of CD161^–^TCRVα7.2^+^ T cells were not significantly different between the two compartments (Fig. 2A, B). However, analogous to the results obtained for the peripheral blood we observed a further reduction of the lymph nodal CD161^+^ MAIT cell frequency in HIV-infected patients to a significantly lower median of 0.06% (IQR = 0.04–0.2%, p = 0.03) in comparison to healthy controls (Fig. 2). A similar reduction of the CD161^+^ MAIT frequency was detectable in all LNMC samples of HIV-infected patients regardless of their treatment status (LNMC from 5 untreated and 6 ART-treated patients were included in our analysis). However, it has to be noted that our analysis was limited by the fact that no paired blood - lymph node samples from the same donors were available.

**Table 2:** Cohort (LNMC samples).

	Healthy	HIV+(high VL and ART)
**number of donors**	6	11
**age [years]**	55 (±16)	44 (±15)
**viral load [copies/ml]**	–	42516 (±58152)

Shown are means and standard deviation.

The frequency of the CD161^–^CD4^–^ T cell subset that expressed TCRVα7.2^+^ but no other MAIT cell-defining markers (e.g. CD161, hence termed: CD161^–^TCRVα7.2^+^ subset), was increased in viremic HIV patients, patients receiving ART and in LTNP (median HC = 0.62%, IQR = 0.44–0.83% vs. median high VL = 1.9%, IQR = 1.0–3.4%, median ART = 1.72%, IQR = 1.46–2.65% and median LTNP = 1.14% IQR = 0.31–4.6%; Kruskal-Wallis test followed by Dunn's multiple comparisons test: p<0.0001, p<0.01 and p<0.0001, respectively) (Fig. 1B middle panel). In the resting state this cell population consists most likely of regular T cells using this Vα chain but their increase in HIV-infected patients might reflect an activation-induced down-regulation of CD161 on the MAIT cell population [13], which was supported by the stable frequency of TCRVα7.2^+^ T cells within the CD3^+^ T cell pool (Fig. 1B, right panel). However, only few untreated highly viremic patients showed very high levels of CD161^–^TCRVα7.2^+^CD4^–^ T cells. A closer analysis revealed that five of these patients were recently diagnosed as HIV positive and at least three of these patients had a documented primary infection with definite seroconversion (<1 month) (Fig. S1A). For these patients, we used the time since HIV diagnosis as a crude measure for the time of infection (Fig. S1B) as previously described [6]. Therefore, it is intriguing to speculate whether this expansion of the CD161^–^TCRVα7.2^+^CD4^–^ T cell subset might be an effect of CD161 down-regulation on former CD161^+^ MAIT cells, taking place during early stages of infection. While in general, there was a great variation between individual subjects in the frequency of MAIT cells and CD161^–^ cells in healthy donors as well as in HIV patients, in HIV patients the MAIT/CD161^–^TCRVα7.2^+^ cell ratio was shifted towards the (expanded) CD161^–^TCRVα7.2^+^ portion (Fig. S1). Of note, in lymph nodes we detected a similar shift of this CD161^+^ MAIT/CD161^–^TCRVα7.2^+^ cell ratio in HIV-infected patients (Fig. 2).

### Functional characterization of CD161^+^ MAIT cells versus CD161^–^TCRVα7.2^+^ cells

MAIT cells are believed to operate by secreting effector cytokines such as TNF-α, IFN-γ as well as IL-17 and IL-22 [1]. In order to assess possible functional impairments of MAIT cells in HIV-infection, we next wanted to explore, whether CD161^+^ MAIT cells and CD161^–^TCRVα7.2^+^CD4^–^ T cells of HIV-infected patients differ in their cytokine secretion profile after unspecific stimulation. Thus, we carried out intracellular cytokine staining assays with PMA/ionomycin-stimulated PBMCs of both groups. Upon PMA/ionomycin-stimulation CD161^+^ MAIT cells produced slightly more IL-17 (p<0.001 and <0.01, for HC and HIV patients respectively) and IFN-γ (p<0.001 for HC, no significant difference in HIV patients) as compared to their CD161^–^TCRVα7.2^+^ counterparts (Fig. 1D). This finding is in line with previous results [8] and suggests that both T cell subsets are functionally different, and that CD161^–^TCRVα7.2^+^ cells are less capable to fulfill Tc17 functionality than CD161^+^ MAIT cells in HIV-infected patients.

### Frequencies of activated CD161^+^ MAIT cells and CD161^–^TCRVα7.2^+^ cells are elevated in the blood of highly viremic HIV patients

**Figure 3:**
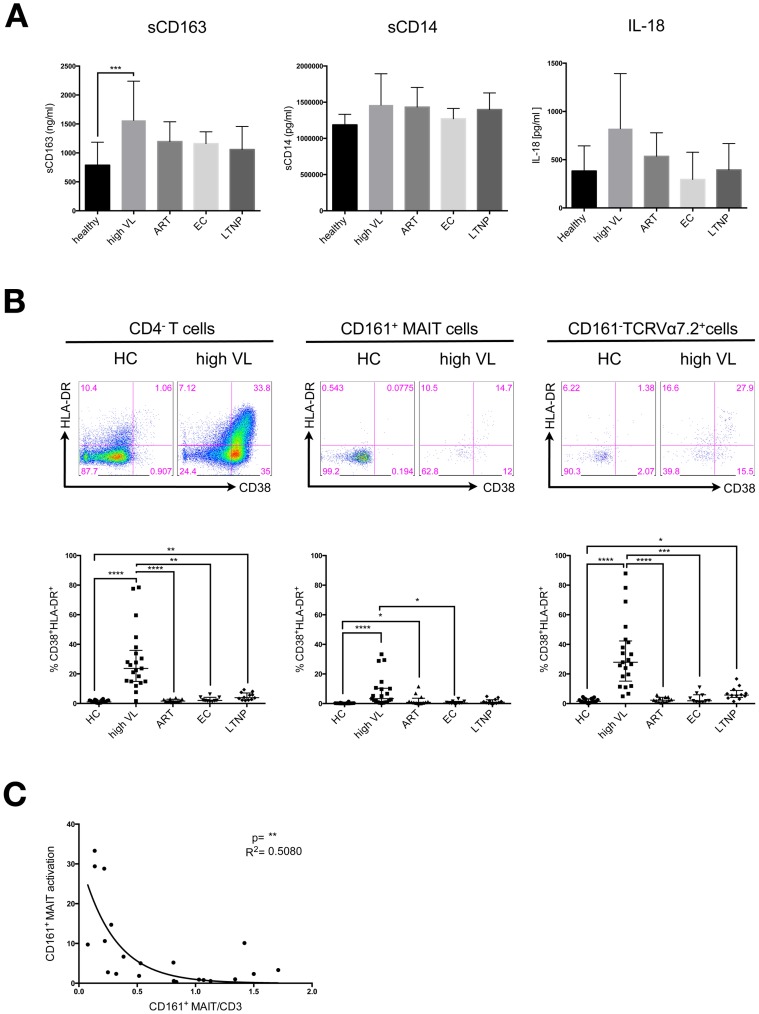
Frequencies of activated CD161^+^ and CD161^–^ TCR Vα7.2^+^ cells are elevated in the blood of highly viremic HIV patients. A) Plasma levels of sCD163, sCD14 and IL-18 were measured by ELISA in mostly corresponding samples of healthy controls (HC n = 13), highly viremic HIV-infected patients (high VL n = 18), patients receiving ART (ART n = 17), elite controllers (EC n = 6) and long-term nonprogressors (LTNP n = 6). B) Activated cells were defined as CD38^+^ and HLA-DR^+^ double positive cells and their frequency was assessed within the CD4^–^, MAIT and CD161^–^TCRVα7.2^+^ populations. PBMC samples were derived from healthy controls (HC), highly viremic HIV-infected patients (high VL), patients receiving ART (ART), elite controllers (EC) and long-term nonprogressors (LTNP). Groups were compared by Kruskal-Wallis test followed by Dunn's multiple comparison test. P-values smaller than 0.05 were considered significant, where *, ** and *** indicate p-values between 0.01 to 0.05, 0.001 to 0.01 and 0.0001 to 0.001 respectively. Bars and lines indicate mean and standard deviation. C) Pearson correlation analysis of CD161^+^ MAIT cell activation and CD161^+^ MAIT cell frequency of CD3^+^ T cells from the untreated high VL patient group. R^2^ quantifies goodness of fit to the non-linear regression (exponential grow equation). R^2^ is a fraction between 0.0 and 1.0, with 1.0 indicating the best fit.

In accordance with previous reports [18], [19] we observed elevated levels of the soluble activation marker sCD163, a monocyte- and macrophage-specific scavenger receptor, shed during activation, in the plasma of untreated patients (Fig. 3A). A similar trend was observed for levels of sCD14, a marker of monocyte response to LPS [20]. However, this trend did not reach statistical significance and was not directly correlated to MAIT cell frequencies (Fig. 3A and data not shown).

In addition to the expression of CD161, MAIT cells are defined by the expression of IL-18R [1]. Therefore, we hypothesized that circulating IL-18 in HIV-infected patients might lead to down-regulation of this receptor as well as to a strong stimulation of these cells. Indeed, IL-18, which is produced by macrophages, dendritic cells and enterocytes, was slightly elevated - though not statistically significant - in the plasma of untreated HIV patients of our cohort when measured in a standard ELISA assay (median high VL = 683.4 pg/ml, IQR = 445.4–1087 pg/ml vs. median HC = 523.4 pg/ml, IQR = 134.2–631 pg/ml) (Fig. 3A). Our results are in line with previous studies that showed that IL-18 is elevated during primary and chronic HIV-infection [21], [22].

We next tested the hypothesis whether potent activation of the CD161^+^ MAIT cell subset could indeed be a possible driving force for MAIT cell depletion. We therefore determined expression of the activation markers HLA-DR and CD38 on the CD161^+^ MAIT cell subset and their parent population (CD4^–^CD3^+^T cells) as well as on the CD161^–^TCRVα7.2^+^ cells (Fig. 3B). The frequency of activated cells within the CD161^+^ MAIT cell subset was significantly elevated in patients with untreated HIV infection and high viral load and slightly in patients receiving ART (median HC = 0.15%, IQR = 0.06–0.28% vs. median high VL = 3.31%, IQR = 0.94–10.35% and median ART = 0.83%, IQR = 0.33–3.63%, Kruskal-Wallis test followed by Dunn's multiple comparisons test: p<0.0001 and p<0.05, respectively). In fact, the activation level of MAIT cells from elite controllers was also significantly lower compared to highly viremic patients (median EC = 0.39%, IQR = 0.12–1.45%; p<0.05).

The CD4^–^ parent population also showed a very high level of activation in viremic patients in comparison to all other patient groups, which indicates that this activation is part of the general immune activation observed in HIV infection and is not specific for the CD161^+^ MAIT cell population (Fig. 3B). However, co-expression of CD38 and HLA-DR on the CD161^+^ MAIT cell subset itself was inversely correlated to the CD161^+^ MAIT cell frequency (Fig. 3C), indicating that at least an indirect effect of activation (for example at early stages of infection) is likely to be associated with the reduction of the CD161^+^ MAIT cell subset.

### *In vitro* stimulation of MAIT cells leads to activation and a reduced CD161^+^ MAIT cell frequency

**Figure 4:**
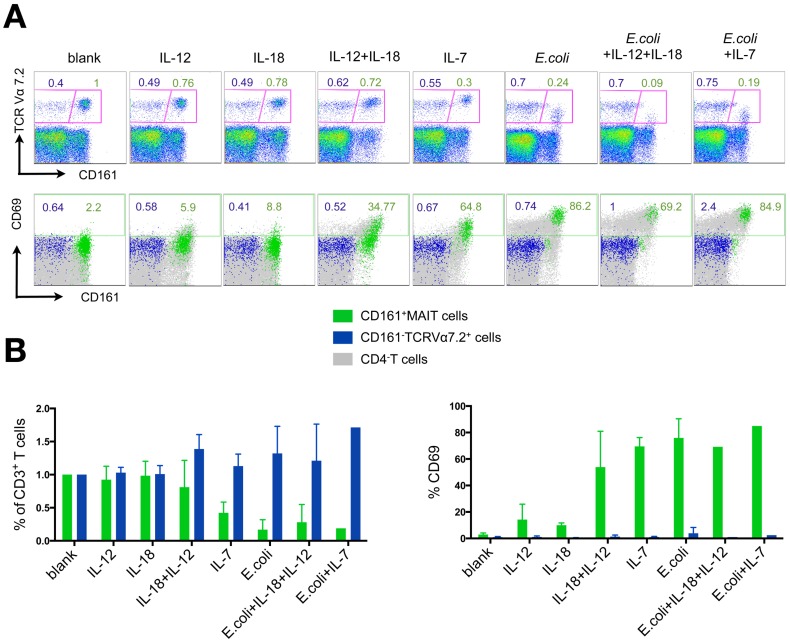
Frequencies of CD161^+^ MAIT cells are reduced upon *in vitro* stimulation with PFA fixed *E.coli*, IL-7 and combined IL-12 and IL-18. **A**) Representative FACS plots depicting frequencies of CD161^–^TCRVα7.2^+^ cells, CD161^+^ MAIT cells and all TCRVα7.2^+^ cells as frequencies of CD3^+^ T cells after 28 hours of stimulation with IL-12 (100 µg/ml), IL-18 (100 µg/ml) and their combination, IL-7 (100 µg/ml) and PFA fixed *E. coli* (bacteria per cell ratio of 100∶1 PBMC). PBMCs were healthy donor-derived and seeded in 1×10^6^ cells/well. **B**) Cumulative display of normalized frequencies of CD161^+^ MAIT cells and CD161^–^TCRVα7.2^+^ cells from 3 healthy donors in duplicate measurements for each stimulation setting (apart from *E.coli* in combination with cytokines, which was tested once) measured after 28 hours of stimulation (left panel). Activation levels were assessed by determining the percentage of CD69^+^ cells within the CD161^+^ MAIT cell and the CD161^–^TCRVα7.2^+^ population.

To determine possible factors that might contribute to the reduction of the CD161^+^ MAIT cell population in HIV-infection on the one hand or possibly could lead to proliferation on the other hand, we tested the effect of cytokines (i.e. cytokines for which MAIT cells highly express the respective receptor) on CD161^+^ MAIT cells. Moreover, the hypothesis of microbial translocation as a driving force for MAIT cell reduction in HIV was tested by adding fixed *E.coli* in a bacteria per cell ratio of 100∶1 to the *in vitro* culture. In an *in vitro* experiment, PBMCs from healthy donors were stimulated with IL-12, IL-18, IL-7, fixed *E.coli* alone or in combination to elucidate the effect on MAIT cell activation (Fig. 4A, B). No significant alteration in the level of activation or frequency of the CD161^+^ MAIT cell population was observed with addition of IL-12 or IL-18 alone (Fig. 4A, B). Combined stimulation with IL-12 and IL-18 in contrast led to potent activation of the MAIT cell subset as measured by CD69 expression (unstimulated cells mean = 2.98±1.16% vs. IL-12+IL-18 mean = 53.88±27.04%) accompanied by a slight reduction of the MAIT cell frequency among CD3^+^ T cells, reflecting synergistic effects of IL-18 and IL-12 (Fig. 4A, B). The reduction of the MAIT cell frequency was confirmed using IL18R or CCR6 expression in addition to CD161 expression as alternative MAIT cell characterization (Fig. S4).

Since IL-7 has been described to be significantly elevated in untreated HIV-Infection [22] and a previous report described IL-7 to be an important cytokine to license MAIT cell effector function [23], the effect of exogenous IL-7 for MAIT cell activation was also tested. In our hands, *in vitro* addition of this cytokine indeed led to the activation of almost the entire CD161^+^ MAIT cell subset. However, this activation was accompanied by a sharp drop of the CD161^+^ MAIT cell frequency (Fig. 4A, B).

Addition of fixed *E. coli* also resulted in pronounced CD161^+^ MAIT cell activation (blank mean = 2.98±1.16% vs. *E. coli* mean = 75.95±14.50%), TCRVα7.2 down-regulation and significant reduction of this cell subset (blank normalized to 1 vs. *E.coli* mean = 0.17±0.15) (Fig. 4A, B).

These results support the hypothesis that the translocation of bacteria and their products as well as consecutively produced pro-inflammatory cytokines (e.g. IL-12, IL-18) contribute to CD161^+^ MAIT cell over-activation and, in turn to their quantitative reduction in HIV-infected patients [7]. Our results are also in line with a recent report that demonstrated that CD161^+^ MAIT cells can be stimulated in an TCR independent manner by the cytokines IL-12 and IL-18 [24].

## Material and Methods

### Study subjects and samples

PBMC as well as lymph node mononuclear cells (derived from hilar and axillary lymph nodes) of in total 63 HIV patients were collected at the University Medical Center Hamburg-Eppendorf and Hannover Medical School, Germany. Healthy individuals (PBMC n = 20, LNMC n = 6) served as controls and for validation of the immunological tests. Written informed consent was obtained from all patients enrolled in this study, which was approved by the Ethics Committee of the Medical Association of Hamburg. The HIV long-term nonprogressor and elite controller cohorts consisted of HIV-infected subjects selected according to viral load, CD4 count, time and duration of HIV infection. The definition of the different stages of the disease were extracted from the electronic databases of the participating centers and confirmed by the treating physicians according to standard classifications and by criteria commonly used in the literature [25], [26]. HIV-1 viral load was determined using COBAS amplicor assays with a detection limit of 50 RNA copies/ml. HIV CDC status, antiretroviral treatment, and CD4^+^ T cell counts were determined via chart review.

### Immunophenotypic analysis

For immunophenotypic staining, cryopreserved PBMC or LNMC were thawed using standardized techniques. For exclusion of dead cells PBMCs were stained with the LIVE/DEAD Fixable Aqua Dead Cell Stain Kit (life technologies, Darmstadt, Germany) according to the manufacturer's protocol. To characterize T cell populations, at least 5×10^5^ cells were stained with appropriate fluorochrome-conjugated surface antibodies, including anti-CD3 (SK7), anti-CD4 (RPAT4), anti-CD8α (HIT8a), anti-TCRVα7.2 (3C10), anti-CD161 (HP-3G10), anti-HLA-DR (G46-6), anti-CD38 (HIT2, Biolegend), anti-IL-18R (H44), anti-CD69 (FN50), anti-CCR6 (G034E3) (from Biolegend or BD Biosciences, Heidelberg, Germany) for 20 min at RT in the dark. After surface staining, cells were washed once with PBS and were then resuspended in 0.5% paraformaldehyde. For intracellular cytokine staining, the cells were stimulated with PMA (50 ng/ml) and ionomycin (0.67 µM) for 5 h at 37°C and 5% CO_2_ prior to immunophenotypic staining. Brefeldin A (10 g/ml) was added for the last 4 h of the stimulation. Data were collected on a BD LSRII machine using FACS Diva version 5 (BD Biosciences, Heidelberg, Germany).

### ELISA of plasma sCD163, sCD14 and IL-18

sCD163 levels in plasma were measured using the Macro163 ELISA kit (Trillium Diagnostics, Bangos, ME). sCD14 levels in plasma were measured using the Human sCD14 Quantikine ELISA kit (R&D Systems, Minneapolis, MN). Plasma levels of IL-18 were measured using the Human IL-18 Platinum ELISA kit (eBioscience, Germany).

### Stimulation of PBMCs with cytokines and fixed *E. coli*

PBMCs from healthy donors were freshly isolated and seeded into a 48 well plate (1×10^6^ cells/well). IL-12, IL-18 and IL-7 were added with an end concentration of 100 ng/ml. Prior to an experiment, aliquots of 1×10^9^ *E.coli* (DH5α)/ml were fixed for 5 min in 1% paraformaldehyde and stored at −80°C. After thawing and washing, *E. coli* was added in a bacteria/cell ratio of 100∶1. The PBMCs were stimulated for either 14 or 28 h before surface staining and FACS analysis.

### Statistical analysis

All flow cytometric data were analyzed using FlowJo version 9.2 software (Treestar). Statistical analysis was carried out using Prism 5.0 software (GraphPad Software, San Diego, CA). All groups were tested for normal distribution with the Kolmogorov-Smirnov test and were compared by the adequate test. For normally distributed data, parametric tests were applied: for two groups t-tests, for more than two groups ANOVA followed by Tukey's multiple comparisons test. Data that was not normally distributed was tested by the Mann-Whitney test for two unpaired groups, by the Wilcoxon test for paired groups, or Kruskal-Wallis test followed by Dunn's multiple comparisons test for more then two groups, respectively. P-values smaller than 0.05 were considered significant, where *, ** and *** indicate p-values between 0.01 to 0.05, 0.001 to 0.01 and 0.0001 to 0.001, respectively. Pearson's correlation was applied for bivariate correlation analyses. Data are expressed as median, 25% and 75% quartiles or means with standard deviation, respectively (as indicated in the figure legend). CD161^+^ MAIT cell frequencies were normalized for the *in vitro* stimulation data.

## Discussion

Only in the last couple of years the important role of CD161^+^TCRVα7.2^+^CD4^–^CD3^+^ MAIT cells in the defense of different bacterial (and indirectly in viral) infections has become clear [1], [2], [6], [12], [24], [27]–[29]. While great progress has been made in characterizing the phenotypic and functional properties of MAIT cells [1], [4], [23], [24], [30]–[33], many details of MAIT cell function still have to be elucidated.

The current study adds further detail to the recent body of data suggesting diminishing function and decreasing numbers of MAIT cells in HIV infection [5]–[7]. In particular, frequency and phenotype of MAIT cells during HIV infection with regards to disease status, including HIV LTNP and elite controllers, were analyzed in detail. In accordance with data from a recent study [6] we confirm an irreversible reduction of the CD161^+^ MAIT cell population in peripheral and lymph nodal tissue of patients with treated and untreated chronic HIV infection. However, while the preliminary results of the study by Leeansyah and coworkers [6] suggests that elite controllers maintain a similar size of their MAIT cell population as healthy controls, we found a significantly reduced MAIT cell frequency, as well as reduced MAIT cell numbers in the ten elite controller patients and a similar trend in twelve long-term nonprogressors included in the present study. These data are potentially of clinical importance and further studies have to elucidate whether the higher general immune activation detected in elite controllers in comparison to healthy controls is in parts due to a lower level of MAIT cells in these patients [34]–[36].

In functional *in vitro* experiments (Fig. 4) we were able to replicate the proposed model of a constitutive down-regulation of functional MAIT cell markers (i.e. CD161, IL-18R, CCR6) and eventual loss of TCRVα7.2^+^CD4^–^CD3^+^ T cells by stimulation with bacterial antigens and pro-inflammatory cytokines in HIV infection [7]. Importantly, we and others found that inflammatory cytokines like IL-12 or IL-18 can activate MAIT cells in a TCR independent manner [24], [37]. It is not quite clear in how far the *in vitro* conditions replicate the *in vivo* milieu, but significant shifts towards inflammatory cytokines have been described and it has been proposed that these cytokines add to the pathogenesis of HIV infection [21], [22].

Clearly, the current study is limited by the fact that no mucosal tissue was available for any of the patients studied and homing of the MAIT cell population to the gut might be in part responsible for the decrease of the peripheral MAIT cell frequencies. However, two independent studies previously showed a loss of mucosal gut resident MAIT cells in HIV-infected patients [5], [6]. In preliminary experiments, we also see high expression of the gut-homing markers CCR9 and beta7 integrin [38] on MAIT cells in the jejunum of healthy controls (data not shown) and up-regulation of CCR9 and beta7 integrin was detected in peripheral MAIT cells in HIV patients (Fig. S3). The frequency of CCR9^+^β7^+^ integrin on MAIT cells was inversely correlated with the frequency of MAIT cells supporting the model of partial homing of MAIT cells to the gut in HIV infection [5]. In future prospective efforts it is planned to analyze the gut resident MAIT cell frequencies in HIV patients with different disease course, including HIV elite controllers obtained during routine screening colonoscopies.

In the present study, we also looked at a small number of patients with a recent history of acute retroviral syndrome, early primary HIV infection and/or confirmed recent seroconversion. In line with the results of the recently published studies [5]–[7], our data indicate that the decrease of peripheral CD161^+^ MAIT cell numbers seems to be an early event in HIV infection. However, very early in the course of the primary infection we find a relative increase of the CD161^–^ TCRVα7.2^+^CD4^–^CD3^+^ T cell subset suggestive of a down-regulation of CD161 on these cells. This elevation can still be detected at later stages and in different patient groups (Fig. 1B, middle panel). These CD161^–^TCRVα7.2^+^CD4^–^CD3^+^ T cells were also recently described [5] and appear to have a larger proportion of cells with a similar TCRVβ usage as seen in MAIT cells in HIV-infected patients. In our hands, these highly activated CD161^–^TCRVα7.2^+^CD4^–^CD3^+^ T cells rapidly develop after *in vitro* stimulation of CD161^+^ MAIT cells and seem to be less functional *ex vivo* than their CD161^+^ MAIT cell counterparts (Fig. 1D). In one patient who is included in our study and was treated during early primary infection, the MAIT cell frequency indeed recovered (Fig. S2). In a next step, further prospective clinical studies of patients with primary HIV infection have to establish whether very early initiation of ART can indeed “rescue” the MAIT cell population [39], [40].

In ongoing experiments we are now characterizing the transcription factor expression profile (RORyt, Tbet, Gata3, PLZF) of CD161^+^ MAIT cells versus CD161^–^ TCRVα7.2^+^CD4^–^CD3^+^ T cells in uninfected donors as well as HIV-infected patients in order to explain some of the detected results of this study and to further discriminate relevant MAIT cell subpopulations [14], [32], [41]–[43]. For example the ability to secrete IL-17 could well be correlated with expression of the transcription factor RORyt [41]. Of importance, recent studies suggest that iNKT cells and MAIT cells exhibit a proapoptotic propensity with elevated expression of activated caspases, which is correlated with expression of transcription factor PLZF/ZBTB-16 [32]. This would make the loss of MAIT cells due to activation-induced cell death in HIV even more plausible. Also, the manipulation of these transcription factors represents an intriguing target for future therapeutic approaches [44]. The proof of “successful genetic manipulation and reprogramming to pluripotency and re-differentiation of functional MAIT cells” opens at least theoretically further therapeutic options [44], [45]. In our hands, it was difficult to culture and expand primary MAIT cells *in vitro* and novel *in vitro* MAIT cell expansion protocols should be developed.

For the time being, it will be important to prospectively examine patients at the first stages of acute HIV infection to understand the exact kinetics of the MAIT cell loss and whether very early initiation of ART during primary HIV infection can indeed rescue the MAIT cell population and thereby possibly minimize subsequent microbial translocation [40]. Other theoretical therapeutic options include the immunomodulatory blockade of circulating cytokines (eg. IL-18) or the use of probiotics to stimulate MAIT cell regeneration and proliferation by activation of the commensal bacterial flora [2], [46]. In a next step, the MAIT cell frequency and function have to be comprehensively examined in different tissues, e.g. lung tissue (via BAL) [28], [47] or the liver [23] of HIV-infected patients (including elite controllers) in comparison to uninfected controls and, importantly, in HIV/TB or HIV/HCV coinfection [47]. Further studies should also comprehensively look at the different immune cell populations with antibacterial activity (e.g. γδ T cells, NKT cells or innate lymphoid cells [48], [49]) as other cells might compensate the loss of the MAIT cell population. For example, we find a relative expansion of γδ T cell subsets in HIV infection ([49] and data not shown). Further analysis of MAIT cells might be assisted by the use of MAIT cell tetramer technology [31]. It will also be interesting to see whether the riboflavin metabolites are in itself more reliable and stable markers for bacterial translocation than LPS [30].

In summary, the current study confirms and extends previous studies and suggests that an irreversible reduction of the peripheral CD161^+^ MAIT as well as an expansion of the CD161^–^TCR Vα7.2^+^ subset are early events in HIV infection [5]–[7], [24], [47]. The loss is most likely caused by a combination of increased homing to the gut as well as a lacking resistance towards stimulation by microbial products and cytokines leading to a) a change of phenotype, b) loss of function and c) possibly cell death, which takes place in all patients including HIV long-term nonprogressors and elite controllers independently of disease progression.

## Supporting Information

Figure S1 **The CD161^+^ MAIT/CD161^–^TCRVα^+^ ratio is shifted in HIV infection.** **A**) Bars indicate each individual's frequency of CD161^+^ MAIT cells (light grey) and CD161^–^TCRVα^+^ cells (dark grey). Patients with confirmed or suspected acute HIV-infection (n = 5) below 1 month post first diagnosis are marked in red. **B**) Comparison of CD4^–^T cell frequencies (grey), CD161^–^TCRVα7.2^+^ cell frequencies (blue), CD161^+^ MAIT cell frequencies (green) of high VL patients below 1 month vs. above 1 month of known HIV-infection. The indicated p-value results from a Mann Whitney test. P-values smaller than 0.05 were considered significant.
(TIF)

Figure S2 **Longitudinal CD161^+^ MAIT cell increase in a patient after early treatment initiation.** FACS plots depicting frequencies of CD161^+^ MAIT (upper right number in green) and CD161^–^TCRVα^+^ cells of the CD4^–^ T cell population (upper left number in blue) of a patient during acute HIV-infection (Fiebig stage III: ELISA +, Immunoblot -) and 28 month later after immediate initiation of ART.
(TIF)

Figure S3 **Migration to the gut is specifically elevated in the CD161^+^ MAIT cell population.** Migrating cells are measured by frequencies of CCR9^+^β7integrin^+^ double positive cells of CD161^+^ MAIT cells and CD161^–^TCRVα7.2^+^ cells, respectively. PBMC samples were derived from healthy controls, highly viremic HIV-infected patients and patients under ART. **A**) Groups were tested for normal distribution by Kolmogorov-Smirnov test and compared by Kruskal-Wallis test followed by Dunn's multiple comparisons test. P-values smaller than 0.05 were considered significant, where *, ** and *** indicate p-values between 0.01 to 0.05, 0.001 to 0.01 and 0.0001 to 0.001 respectively. Bars and lines indicate median and interquartile ranges. **B**) Correlation analysis of CD161^+^ MAIT cell frequency or CD161^–^TCRVα7.2^+^ cell frequency with the corresponding frequency of CCR9^+^/β7^+^ CD161^+^MAIT cells. R^2^ is a fraction between 0.0 and 1.0, with 1.0 indicating the best fit to the linear regression.
(TIF)

Figure S4 **The MAIT cell defining markers CD161, IL18R and CCR6 are reduced within the TCRVα7.2^+^ subset upon stimulation with IL-12 and IL-18, IL-7 and** ***E.coli*** **.** **A**) Representative FACS plots depicting frequencies of CD161^–^TCRVα7.2^+^ cells, CD161^+^ MAIT cells and all TCRVα7.2^+^ cells as frequencies of CD3^+^ T cells after 28 hours of stimulation with IL-12 (100 µg/ml), IL-18 (100 µg/ml) and their combination, IL-7 (100 µg/ml) and PFA fixed *E. coli* (bacteria per cell ratio of 100∶1 PBMC). PBMCs were healthy donor-derived and seeded in 1×10^6^ cells/well.
(TIF)
