# Supplementary figures and images for: CD161+ MAIT Cells Are Severely Reduced in Peripheral Blood and Lymph Nodes of HIV-Infected Individuals Independently of Disease Progression
Source: PLoS One. 2014 Nov 4;9(11):e111323. doi: 10.1371/journal.pone.0111323 (PMC4219715; doi:10.1371/journal.pone.0111323)

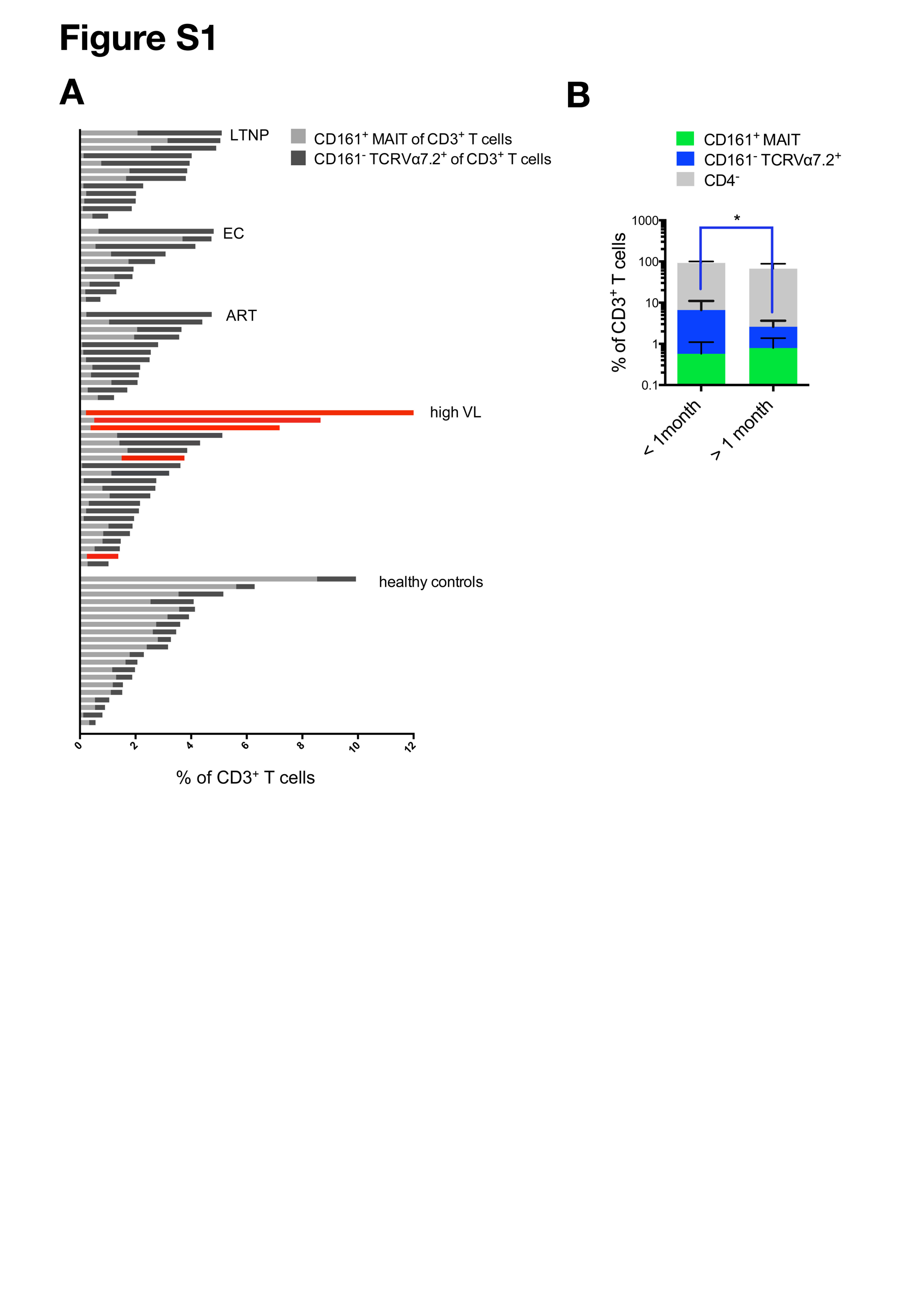

Supplement: Figure S1 — The CD161+ MAIT/CD161–TCRVα+ ratio is shifted in HIV infection. A) Bars indicate each individual's frequency of CD161+ MAIT cells (light grey) and CD161–TCRVα+ cells (dark grey). Patients with confirmed or suspected acute HIV-infection (n = 5) below 1 month post first diagnosis are marked in red. B) Comparison of CD4–T cell frequencies (grey), CD161–TCRVα7.2+ cell frequencies (blue), CD161+ MAIT cell frequencies (green) of high VL patients below 1 month vs. above 1 month of known HIV-infection. The indicated p-value results from a Mann Whitney test. P-values smaller than 0.05 were considered significant. (TIF) [file pone.0111323.s001.tif]

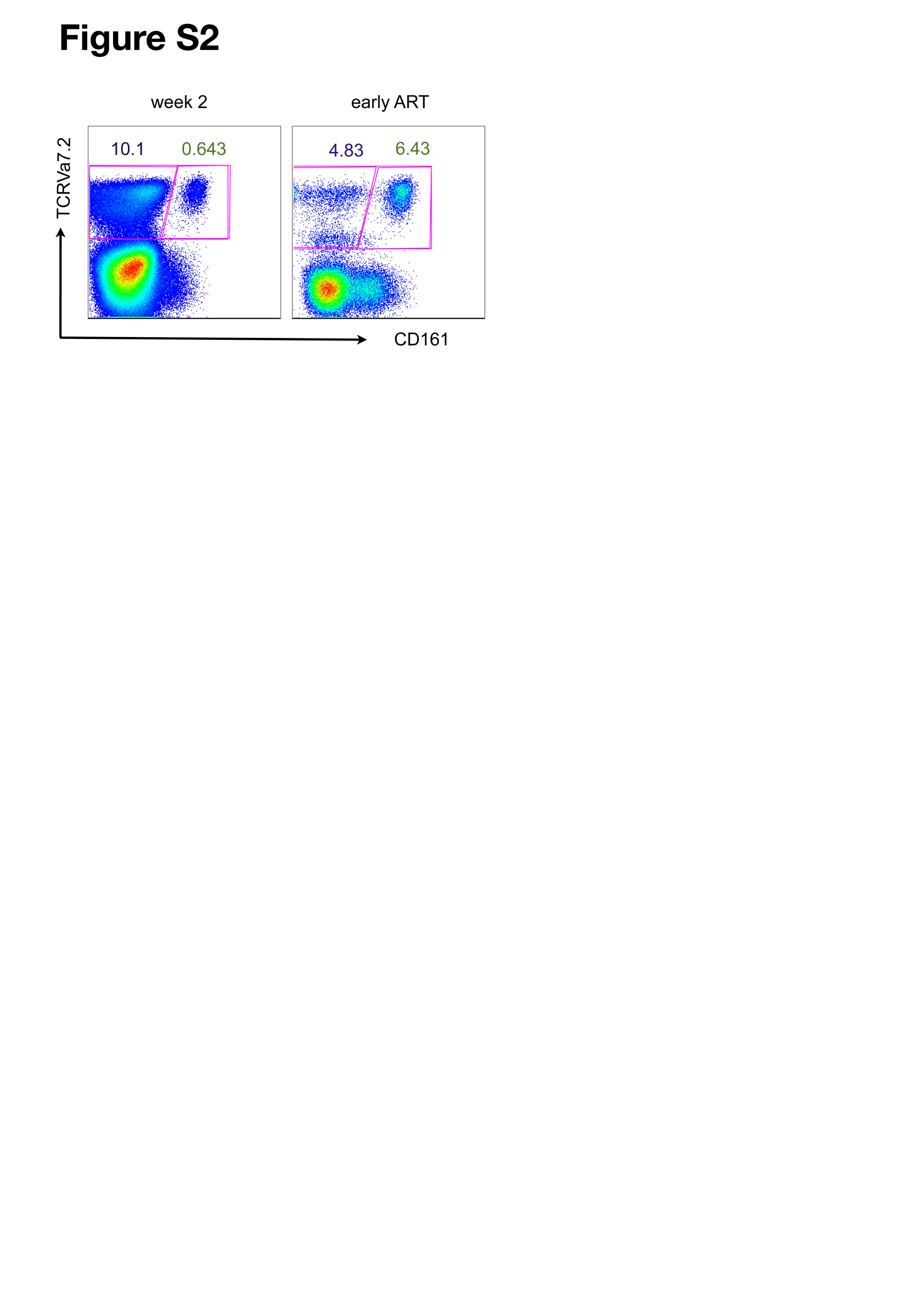

Supplement: Figure S2 — Longitudinal CD161+ MAIT cell increase in a patient after early treatment initiation. FACS plots depicting frequencies of CD161+ MAIT (upper right number in green) and CD161–TCRVα+ cells of the CD4– T cell population (upper left number in blue) of a patient during acute HIV-infection (Fiebig stage III: ELISA +, Immunoblot -) and 28 month later after immediate initiation of ART. (TIF) [file pone.0111323.s002.tif]

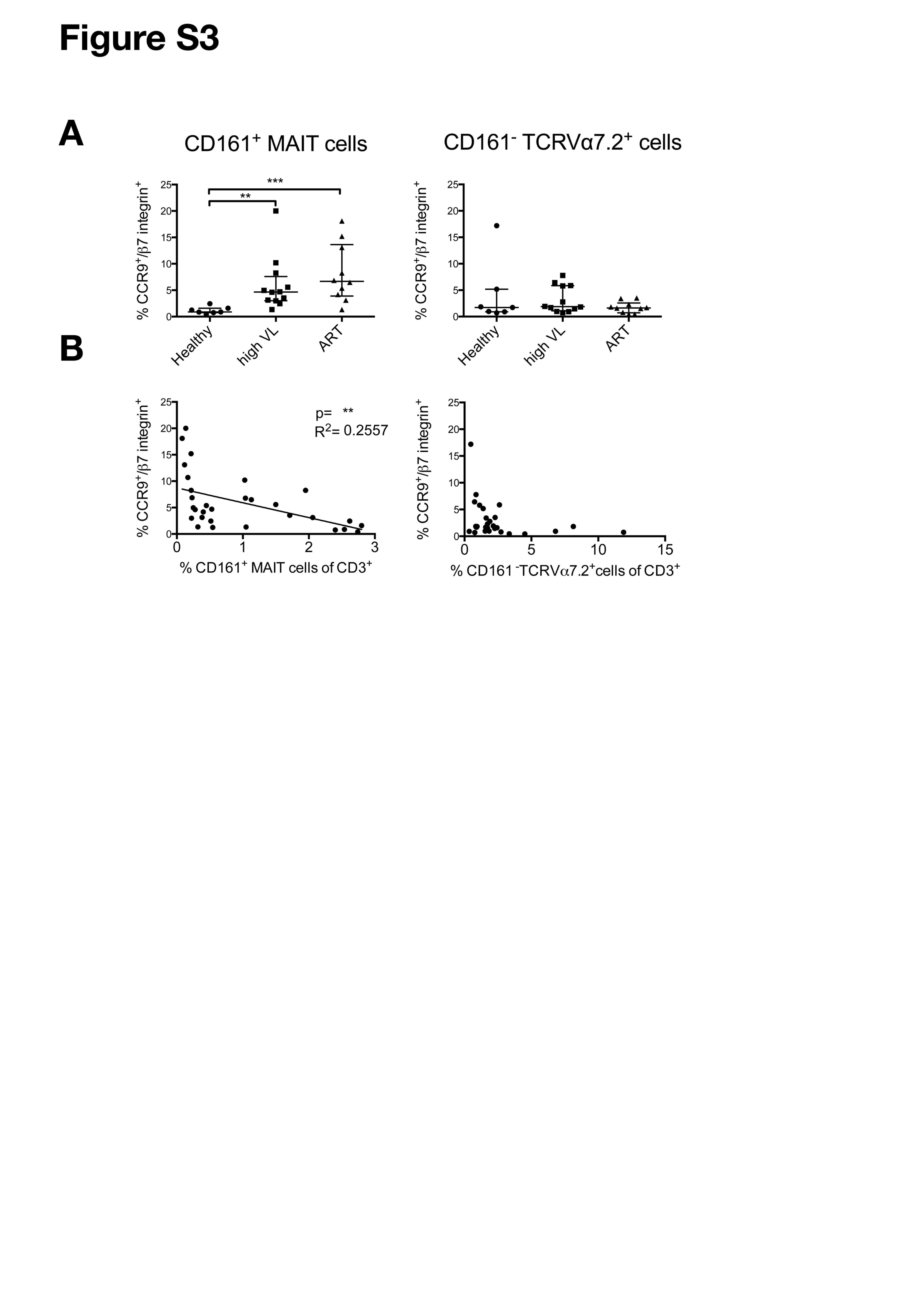

Supplement: Figure S3 — Migration to the gut is specifically elevated in the CD161+ MAIT cell population. Migrating cells are measured by frequencies of CCR9+β7integrin+ double positive cells of CD161+ MAIT cells and CD161–TCRVα7.2+ cells, respectively. PBMC samples were derived from healthy controls, highly viremic HIV-infected patients and patients under ART. A) Groups were tested for normal distribution by Kolmogorov-Smirnov test and compared by Kruskal-Wallis test followed by Dunn's multiple comparisons test. P-values smaller than 0.05 were considered significant, where *, ** and *** indicate p-values between 0.01 to 0.05, 0.001 to 0.01 and 0.0001 to 0.001 respectively. Bars and lines indicate median and interquartile ranges. B) Correlation analysis of CD161+ MAIT cell frequency or CD161–TCRVα7.2+ cell frequency with the corresponding frequency of CCR9+/β7+ CD161+MAIT cells. R2 is a fraction between 0.0 and 1.0, with 1.0 indicating the best fit to the linear regression. (TIF) [file pone.0111323.s003.tif]

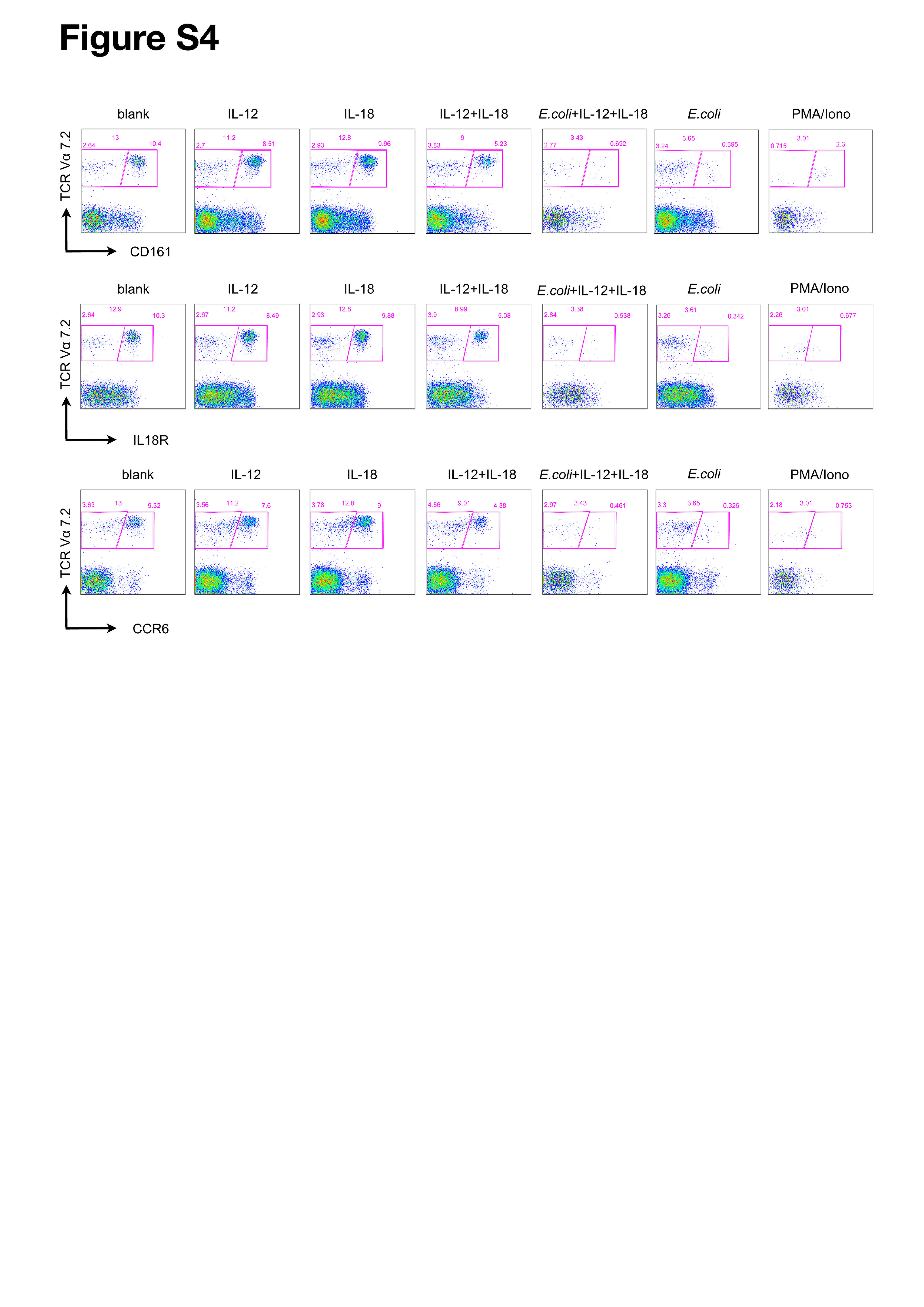

Supplement: Figure S4 — The MAIT cell defining markers CD161, IL18R and CCR6 are reduced within the TCRVα7.2+ subset upon stimulation with IL-12 and IL-18, IL-7 and E.coli . A) Representative FACS plots depicting frequencies of CD161–TCRVα7.2+ cells, CD161+ MAIT cells and all TCRVα7.2+ cells as frequencies of CD3+ T cells after 28 hours of stimulation with IL-12 (100 µg/ml), IL-18 (100 µg/ml) and their combination, IL-7 (100 µg/ml) and PFA fixed E. coli (bacteria per cell ratio of 100∶1 PBMC). PBMCs were healthy donor-derived and seeded in 1×10^6^ cells/well. (TIF) [file pone.0111323.s004.tif]
